# Organic Delusional Disorder in the Context of Huntington’s Disease (ICD 11 F06.2): A Case Report

**DOI:** 10.1192/bjo.2025.10707

**Published:** 2025-06-20

**Authors:** Segun Ayanda

**Affiliations:** Kent and Medway Partnership Trust, Maidstone, United Kingdom

## Abstract

**Aims:** Miss X, a 39-year-old postpartum mother with a strong family history and previous diagnosis of Huntington’s disease, was admitted to mother and baby unit three months after delivery due to psychotic symptoms and delusional disorder. Initially admitted informally, her deteriorating mental state necessitated detention under Section 2 of the Mental Health Act. Her symptoms significantly impaired her ability to care for herself and her children.

**Methods:** Her psychiatric history included past depression and anxiety, previously managed with counselling, but no prior inpatient admissions. Family history was significant for Huntington’s disease in her mother, maternal aunt, and grandfather, as well as schizophrenia in her paternal aunt.

Miss X presented with persistent paranoid delusions, believing she was being stalked by both a male and female figure whom she could see and hear throughout the day. These hallucinations interfered with her sleep, appetite, and self-care. Additionally, she reported intrusive thoughts of accidentally harming her baby, further affecting her ability to meet the needs of her children. She lacked insight into her condition and was treated with risperidone for her psychotic symptoms and mirtazapine for depressive symptoms, given the history of Huntington’s disease and the distress following the loss of custody of her baby.

Investigations revealed no acute intracranial abnormality on CT Head, but there was mild diffuse cerebral and hippocampal atrophy disproportionate for her age. Cognitive assessments included a Frontal Assessment Battery score of 16/18 and an ACE-III score of 78/100. Urine dip on admission was negative. Blood investigations and ECG were largely unremarkable. Differential diagnoses considered included Post-partum psychosis, Schizophrenia, Schizophreniform disorder.

**Results:** Miss X’s lack of insight into her Huntington’s disease has contributed to a significant breakdown in her caregiving ability. The persistence of her paranoid delusions, particularly the belief of being stalked, suggests a deeply entrenched belief system that may have been reinforced over time, making it more resistant to treatment. The overlap between neurodegenerative and psychiatric symptoms presents a challenge in management, requiring a tailored, multidisciplinary approach.

**Conclusion:** This case highlights the complex interplay between Huntington’s disease and severe psychiatric manifestations, particularly delusional disorder. Her enduring psychotic symptoms, compounded by cognitive impairment, significantly impact her functionality and caregiving capacity. Early intervention, close psychiatric monitoring, and integrated neurological care are essential to improve patient outcomes.

